# A Case of Acute Eosinophilic Pneumonia Associated With Non-steroidal Anti-inflammatory Drugs

**DOI:** 10.7759/cureus.52159

**Published:** 2024-01-12

**Authors:** Rui Ribeiro, Andreia Teixeira, Tânia Lopes, Célia Cruz

**Affiliations:** 1 Internal Medicine, Centro Hospitalar Universitário de Santo António, Porto, PRT; 2 Internal Medicine, Unidade Local de Saúde do Norte Alentejano, Hospital de Santa Luzia, Elvas, PRT

**Keywords:** celecoxib, naproxen, eosinophilic pneumonias, non-steroidal antiinflammatory drugs, drug induced eosinophilic pneumonia

## Abstract

Non-steroidal anti-inflammatory drugs (NSAIDs) are widely used and their gastric, cardiovascular, and renal adverse effects have been well documented. Although rare, NSAID-induced acute eosinophilic pneumonia (AEP) may occur. We report a case of AEP related to naproxen and celecoxib. The patient presented with dry cough and breathlessness two weeks after she started taking these drugs. The chest radiograph displayed bilateral opacities and she had peripheral eosinophilia. Bronchoalveolar lavage was performed at a time when blood eosinophilia was already decreasing and cell analysis revealed 63700 cells/mL with 9% eosinophil. After ruling out other possible etiologies, drug-induced AEP was diagnosed. The patient improved after drug discontinuation. When it comes to drug-induced AEP identifying a causative agent is essential as cessation of the drug is the mainstay of the treatment.

## Introduction

Non-steroidal anti-inflammatory drugs (NSAIDs) are used worldwide in the treatment of acute pain and their gastric, cardiovascular, and renal adverse effects have been well documented [1]. Some examples of these effects are peptic ulcer disease, exacerbation of heart failure, and acute kidney injury [1].

Acute eosinophilic pneumonia (AEP) was first described in 1989 as an acute febrile illness with hypoxemia, diffuse pulmonary infiltrates, and bronchoalveolar lavage (BAL) eosinophilia [2]. Though mostly idiopathic, various offending agents have been identified including tobacco smoke, toxins, infections, and drugs [3]. Among drugs, antibiotics are the most commonly reported [4]. Some case reports of naproxen-induced AEP have been published [5] but to our best knowledge, this is the second reported case of a patient taking celecoxib and presenting with eosinophilic pneumonia, enhancing this possible association [6]. 

## Case presentation

A 77-year-old woman with a medical history of osteoporosis and chronic gastritis presented to the emergency department with one-week-old complaints of dry cough and breathlessness. Two weeks before symptom onset the patient started taking celecoxib and naproxen pills for bilateral shoulder pain. She denied tobacco and other inhaled drugs use as well as taking herbal supplements and travels outside Portugal the previous year. The patient reported the appearance of an urticariform skin rash when exposed to topical diclofenac in the past. On her physical examination, she was febrile (38.2ºC tympanic temperature) and bilateral crackles were audible in pulmonary auscultation. There was no rash or wheezing. Hypoxemic respiratory failure with a ratio of partial pressure of oxygen in arterial blood to the fraction of inspiratory oxygen concentration (PaO2/FiO2) of 257 was also identified. The patient was admitted for further investigation and treatment.

The blood tests revealed eosinophilia, elevated C-reactive protein, and sedimentation rate (Table 1).

**Table 1:** Laboratory studies upon admission.

Laboratory finding	Reference range	Admission
Leucocytes, cells x10^3^/uL	4-11	12.07
Neutrophils cells x10^3^/uL	2-7.5	7.43
Lymphocytes cells x10^3^/uL	1.5-4	3.05
Monocytes cells x10^3^/uL	0.2-0.8	0.72
Eosinophils, cells x10^3^/uL	0.04-0.4	0.85
Basophils cells x10^3^/uL	0.02-0.1	0.02
Erythrocyte sedimentation rate, mm/h	0-35	135
C-reactive protein, mg/L	0.5-5	320.96
Creatinine, mg/dL	0.5-0.9	0.63
Urea, mg/dL	20-50	45

**Figure 1:**
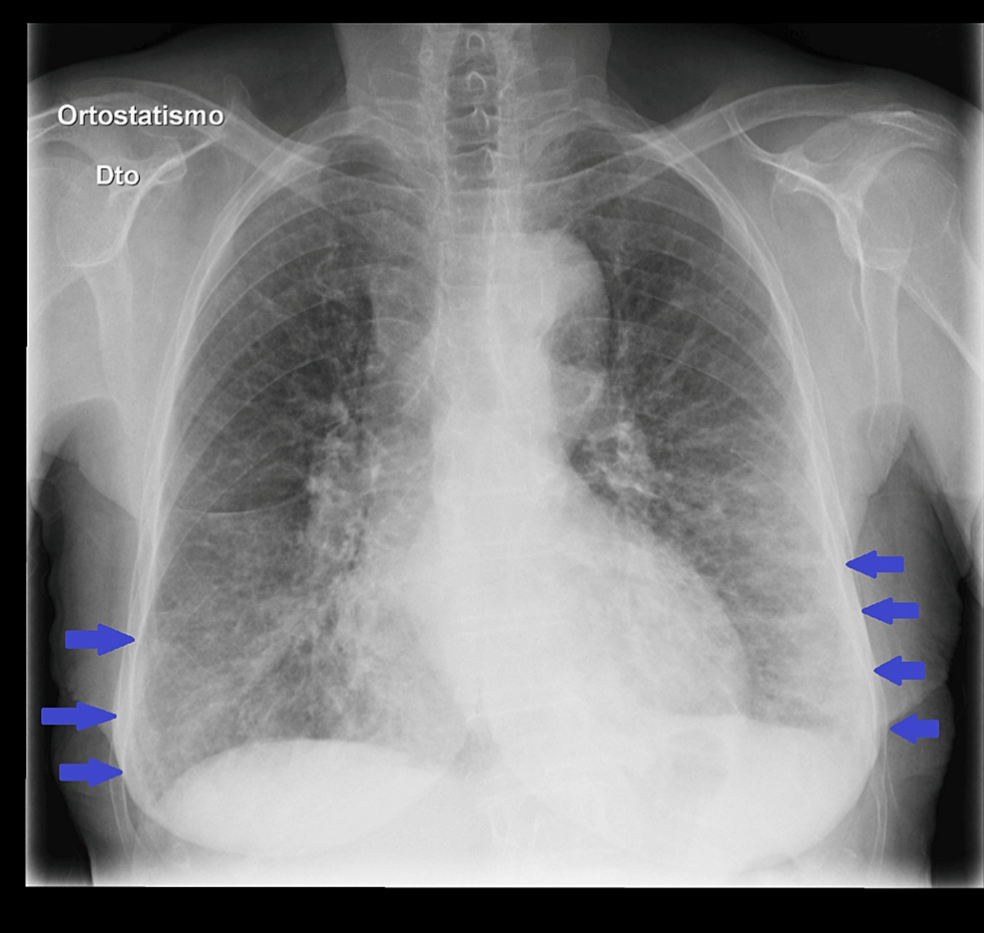
Chest radiograph upon admission showing bilateral subpleural opacities, highlighted by the blue arrows.

The chest radiograph (Figure 1) showed bilateral subpleural opacities predominantly in the lower lobes.

**Figure 2:**
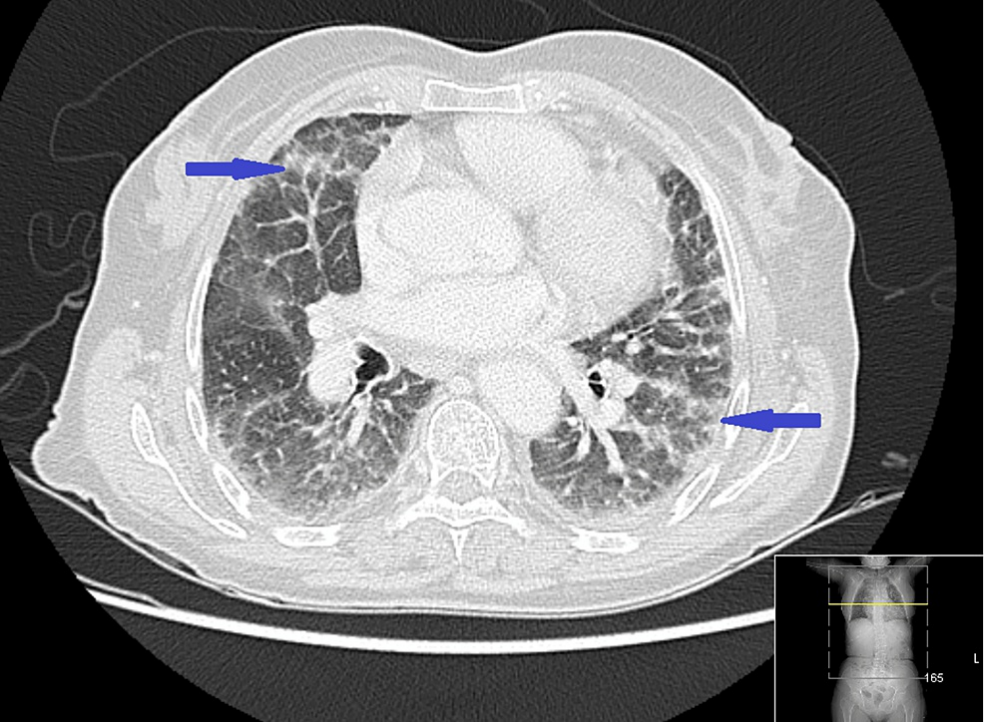
Computed tomography of the thorax showing bilateral peripheral areas of lung consolidation, highlighted by the blue arrows.

Computed tomography (CT) of the thorax showed bilateral peripheral areas of lung consolidation (Figure 2).

HIV serology, antinuclear antibodies (ANA), and antineutrophil antibodies (ANCA) were all negative. A bronchoscopy with BAL was performed 12 days after admission due to logistical difficulties. The BAL specimen was cellular, with eosinophilic alveolitis (63700 cells/mL with 9% eosinophil), with no other cells above normal ranges. This exam was performed at a time when blood eosinophilia had fallen to 0,34 x 10^3^ cells/µL. The bacterial, fungal, and mycobacterial cultures were negative. A nasal swab polymerase chain reaction viral test was made upon admission. It was negative for respiratory syncytial virus; influenza A, B; parainfluenza 1, 2, 3, and 4; adenovirus; metapneumovirus; bocavirus; enterovirus; rinovirus; and coronavirus. 

**Figure 3:**
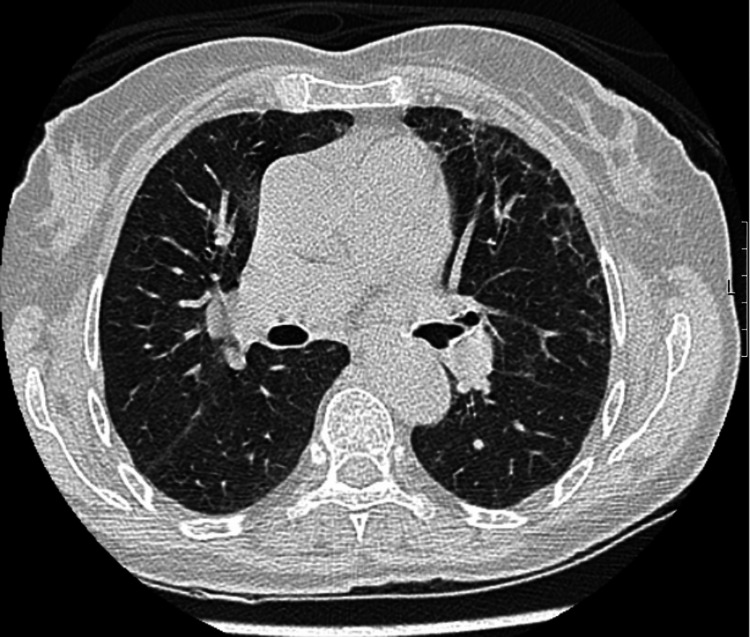
Computed tomography of the thorax showing improvement of the airspace consolidations.

Celecoxib and naproxen were suspended upon admission. The patient showed continuous analytical and clinical improvement and it was decided not to initiate corticosteroid therapy. She remained in hospital for 18 days and was submitted to a revaluation CT 10 days after the first one with significant improvement (Figure 3). The patient showed complete resolution of peripheral eosinophilia and was symptom-free at discharge. It was decided not to do a rechallenge test and the patient was advised to avoid NSAIDs.

The patient complied with NSAID avoidance and completed a two-year outpatient follow-up without symptoms or peripheral eosinophilia recurrence.

## Discussion

Eosinophilia can be defined as a raised eosinophilic count of ≥ 0,5 x 10^3^ cells/μL. Eosinophilic lung diseases are characterized by the presence of peripheral blood eosinophilia and increased eosinophils in BAL fluid or eosinophilic infiltration of the lung demonstrated on biopsy [3]. These can be divided into two major groups: systemic and lung-limited disorders. This patient had an unremarkable immunologic screen test, no history of recent trips to tropical areas of the globe, and no clinical findings suggesting other organ involvement besides the lung. Therefore, taking into account the acute clinical setting and drug expositions, the diagnosis of secondary AEP was made.

AEP is a rare disease with an estimated incidence of approximately 10 per 100,000 person-years [3].

Initial reports were mostly idiopathic. In recent years, multiple causes, most importantly tobacco smoke and several drugs, were identified as causative agents [3]. Although the clinical syndrome known as AEP was first described in 1989 [2], there are some reports that describe an acute febrile illness with pulmonary infiltrates and peripheral/alveolar eosinophilia secondary to naproxen therapy previous to that [7,8]. A literature review of NSAID-induced AEP published in 2018 [5] stated that naproxen was the most common drug of this group associated with this entity. In that same article, there were no reports of celecoxib-induced AEP [5]. Authors found one previous case report linking celecoxib and eosinophilic pneumonia [6].

The pathogenesis of AEP and mainly drug-induced AEP is not completely understood. It is hypothesized that antigenic stimulation of T helper 2 lymphocytes with subsequent production of interleukin-5 and eosinophil recruitment may be one of the mechanisms involved [9].

Our patient reported a previous cutaneous urticariform reaction to topical NSAID. There was no description of hypersensitivity reactions to NSAIDs in previous reports but some patients reported previous allergic reactions to antibiotics [8]. The relevance of this finding is unclear.

Currently, modified Philit criteria [10] are used to diagnose AEP and are as follows: a) acute respiratory illness with 1 month or less duration; b) pulmonary infiltrates on chest radiography; c) hypoxemia (partial pressure of oxygen of less than 60 mmHg or pulse oximetry reading of < 90% on room air); d) pulmonary eosinophilia as demonstrated by 25% or more eosinophils in BAL fluid or eosinophilic pneumonia in lung biopsy; e) absence of evidence of infection or other known causes of eosinophilic lung disease.

Solomon and Schwarz published a paper on drug, toxin, and radiation therapy-induced eosinophilic pneumonia and reported that antibiotics and NSAIDs are the most related drugs [11]. They proposed five criteria to diagnose drug-induced eosinophilic pneumonia: a) the presence of simple, acute, or chronic eosinophilic pneumonia by current diagnostic criteria; b) the presence of a likely or potential candidate drug in an appropriate time frame; c) exclusion of other causes of eosinophilic pneumonia (parasites, fungal infection); d) clinical improvement after cessation of the suspect drug or toxin; e) recurrence of eosinophilic pneumonia with rechallenge to the drug or toxin. In clinical practice, however, rechallenge is unnecessary and potentially dangerous.

This case presented all diagnostic criteria for AEP, except BAL eosinophilia percentage. The cornerstone of treating drug-related AEP is drug withdrawal [4], with or without corticosteroid adjuvant therapy. After patient admission, eosinophilic lung-related drugs were withdrawn, namely NSAIDs, and clinical improvement, with no corticosteroid therapy, was achieved. We believe that performing BAL after 12 days of drug suspension could justify a lower percentage of eosinophilic alveolitis. The threshold of 25% is noted to be diagnostic of eosinophilic pneumonia in the American Thoracic Society Clinical Practice Guidelines too [12]. However, the ≥25% cut-off may not always be applicable in drug-induced AEP [13]. It was been suggested that substituting ≥25% BAL eosinophils for an abnormal percentage of BAL eosinophils by each institution's standards may be more suitable [13]. Our institution's normal range is <1%.

In this scenario, there were two possible offending agents: naproxen and celecoxib. They were withdrawn at the same time making it impossible to determine which drug had led to AEP. Because of a higher number of reports of naproxen-induced AEP [5], we believe that it is the most likely causative agent. A rechallenge would be essential to figure out which of the NSAIDs was responsible for the illness but we considered that the potential benefit did not outweigh the potential harm.

Taking into account that this patient had developed urticaria when exposed to diclofenac and now presented with a delayed hypersensitivity reaction to two other NSAIDs, the authors hypothesize that she might have a class hypersensitivity instead of a specific NSAID sensitivity. Other delayed reactions include aseptic meningitis and nephritis [14].

The definitive treatment of any drug-induced condition is discontinuation of the offending agent. Corticosteroids induce apoptosis of eosinophils and hasten resolution but its use should be dictated by the patient's condition.

## Conclusions

AEP should be suspected in the clinical setting of a patient with acute fever and respiratory symptoms, peripheral eosinophilia, and radiographic pulmonary infiltrates. Drug-induced AEP is a rare condition that requires careful history taking and a high level of suspicion. Identifying a causative agent is essential as its removal is the cornerstone of therapy. NSAIDs are among the most common drugs associated with AEP.

Reporting drug reactions is essential to increase knowledge and guarantee a broad view of the pros and cons prior to prescription.

## References

[1] Bindu S, Mazumder S, Bandyopadhyay U (2020). Non-steroidal anti-inflammatory drugs (NSAIDs) and organ damage: a current perspective. Biochem Pharmacol.

[2] Allen JN, Pacht ER, Gadek JE, Davis WB (1989). Acute eosinophilic pneumonia as a reversible cause of noninfectious respiratory failure. N Engl J Med.

[3] De Giacomi F, Vassallo R, Yi ES, Ryu JH (2018). Acute eosinophilic pneumonia. Causes, diagnosis, and management. Am J Respir Crit Care Med.

[4] Bartal C, Sagy I, Barski L (2018). Drug-induced eosinophilic pneumonia: a review of 196 case reports. Medicine (Baltimore).

[5] Krabansky F, Azzouz B, Biya J, Abou Taam M, Morel A, Trenque T (2018). Eosinophilic pneumonia induced by non-steroidal anti-inflammatory drugs: an underestimated risk. Therapie.

[6] Mehandru S, Smith RL, Sidhu GS, Cassai N, Aranda CP (2002). Migratory pulmonary infiltrates in a patient with rheumatoid arthritis. Thorax.

[7] Nader DA, Schillaci RF (1983). Pulmonary infiltrates with eosinophilia due to naproxen. Chest.

[8] Buscaglia AJ, Cowden FE, Brill H (1984). Pulmonary infiltrates associated with naproxen. JAMA.

[9] Allen JN (2004). Drug-induced eosinophilic lung disease. Clin Chest Med.

[10] Philit F, Etienne-Mastroïanni B, Parrot A, Guérin C, Robert D, Cordier JF (2002). Idiopathic acute eosinophilic pneumonia: a study of 22 patients. Am J Respir Crit Care Med.

[11] Solomon J, Schwarz M (2006). Drug-, toxin-, and radiation therapy-induced eosinophilic pneumonia. Semin Respir Crit Care Med.

[12] Meyer KC, Raghu G, Baughman RP (2012). An official American Thoracic Society clinical practice guideline: the clinical utility of bronchoalveolar lavage cellular analysis in interstitial lung disease. Am J Respir Crit Care Med.

[13] Phillips J, Cardile AP, Patterson TF, Lewis JS 2nd (2013). Daptomycin-induced acute eosinophilic pneumonia: analysis of the current data and illustrative case reports. Scand J Infect Dis.

[14] Kowalski ML, Makowska JS, Blanca M (2011). Hypersensitivity to nonsteroidal anti-inflammatory drugs (NSAIDs) - classification, diagnosis and management: review of the EAACI/ENDA(#) and GA2LEN/HANNA*. Allergy.

